# Roles of Indulgence versus Restraint Culture and Ability to Savor the Moment in the Link between Income and Subjective Well-Being

**DOI:** 10.3390/ijerph19126995

**Published:** 2022-06-07

**Authors:** Bin Li, Sijun Wang, Xinyue Cui, Zhen Tang

**Affiliations:** 1 School of Management, Jinan University, Guangzhou 510632, China; cxyuepsy@163.com; 2 The Institute of Enterprise Development, Jinan University, Guangzhou 510632, China; 3 Department of Marketing and Business Law, Loyola Marymount University, Los Angeles, CA 60045, USA; zhen.tang@lmu.edu

**Keywords:** subjective well-being, financial satisfaction, a sense of control, indulgence versus restraint, ability to savor the moment

## Abstract

Over the past few decades, various academic fields have reported contradictory findings regarding whether income is positively or negatively associated with subjective well-being (SWB). To reconcile the inconclusive observations, researchers tend to use various mediators and moderators that could explain why income is more strongly associated with SWB for certain groups of people and why income could be negatively associated with SWB for other groups. This study endeavored to first test additional mediation roles of financial satisfaction and a sense of control in the link between income and three related yet distinct measures of SWB: life satisfaction, happiness, and emotional well-being (EWB), at both cross-national and individual levels. We further investigated the direct and moderating impacts of national difference in Indulgence versus Restraint (IVR) cultural orientations over two mediating mechanisms (income → financial satisfaction → SWB; income → a sense of control → SWB) using data from 49,097 participants in the 2017–2020 World Values Survey. Additionally, we conducted a moderated mediation analysis of individual difference in ability to savor the moment (ASM) for these two mediating mechanisms based on surveys with 796 respondents from China. Analyses at both national level and individual level confirmed the partial mediating roles of financial satisfaction and a sense of control. We further find a positive, direct impact of IVR on SWB such that people in more indulgence cultures report a higher SWB than those in more restraint cultures. The mediating effects of financial satisfaction were found to be weaker in more indulgence cultures than in more restraint ones, while the mediating effects of a sense of control remain the same. Finally, we find that individuals’ ASM does not only directly lead to a higher SWB, but also amplifies the mediation impact of financial satisfaction in the link between income and life satisfaction and in the link between income and EWB. Implications of these findings are offered for public policy makers, employers, and citizens, as well as researchers from different fields.

## 1. Introduction

As a universally cherished goal, subjective well-being (SWB), defined as the affective and cognitive evaluation of life [1,2], has attracted numerous studies to identify various predictors of SWB in the past few decades. Among all the interesting discoveries, the link between income (and other socioeconomic status) and SWB is among the most intriguing. On the one hand, previous studies reported varied strength sizes of the positive association [3,4]. On the other hand, some studies found a weak positive or no association between income and SWB [5,6,7]. We have also witnessed some seemingly negative associations between income and SWB according to the 2019 World Happiness Report [8]. To reconcile the inconclusive findings regarding the link between income and SWB and some conflicting observations, previous researchers directed their attention to key mediators and moderators that could explain why income is more strongly associated with SWB for certain groups of people and why income could be negatively associated with SWB for other groups.

From the mediator approach, financial satisfaction and a sense of control were among the most recently reported mediators [9,10,11]. By definition, financial satisfaction reflects the value of actual financial resources (e.g., income, salary, assets) relative to one’s desires and expectations for financial resources and one’s actual financial status [12]. A sense of control was defined with two dimensions: personal mastery and perceived constraints [13]; personal mastery refers to “one’s sense of efficacy or effectiveness in carrying out goals” while perceived constraints measure “to what extent one believes there are obstacles or factors beyond one’s control that interfere with reaching goals” [13]. The mediation roles of financial satisfaction and a sense of control in the link between income and SWB (i.e., income → financial satisfaction → SWB; income → a sense of control → SWB) were confirmed at the individual level [9,13,14]. To our best knowledge, no cross-national level analyses were reported to confirm these two mediation mechanisms. For public policy makers, investigating the mediation roles of financial satisfaction and a sense of control is critical because (dis)confirmations of such relationships at the cumulative level would point them in different directions.

From the moderator approach, researchers had proposed two groups of moderators, national differences and personal factors, to help reconcile the perplexed association between income and SWB. National differences include socioeconomic contexts such as popularity density, income inequality, and social mobility [15,16], cultural values such as individualism versus collectivism, uncertainty avoidance, and masculinity versus femininity [17,18,19], and political factors [20,21], among others. In terms of personal factors, researchers have found that the direction and strength of the link between income and SWB differ by gender [22], age [23], personality [24], and social class memberships [13].

While all these moderators are useful, two important variables were neglected by the current literature. The first factor is the newly added sixth dimension of cultural orientations in Hofstede’s framework [25]—indulgence versus restraint (IVR) cultural orientations. By definition, indulgence cultures allow relatively free gratification of basic and natural human desires related to enjoying life and having fun while restraint cultures control gratification of needs and regulate people’s gratification of human needs by means of strict social norms [17]). As a newly added dimension of cultural orientations, IVR’s impact on SWB has not yet received much attention in the current literature.

The other overlooked individual variable is ASM, people’s ability to obtain and savor positive events [26,27,28]. Because the majority of individual factors that have been proposed to moderate the association between income and SWB are hard to change by individuals, exploring the moderating effects of ability-based variables such as ASM promises more opportunities for people to take action toward higher SWB via acquiring and honing their ASM abilities. Some scattered evidence seems to suggest a positive relationship between ASM and SWB [26]; little is known about how ASM could moderate the strengths or directions of the link between income and SWB [29].

To fill the above identified gaps in the current SWB literature, this research first seeks to propose and test partial mediation roles of financial satisfaction and a sense of control at both cross-national and individual levels. Based on two studies, we conducted a moderated mediation analysis of national difference in IVR cultural orientations and individual difference in ASM for these two mediating mechanisms.

## 2. Literature Review and Hypotheses Development

### 2.1. Indices of Subjective Well-Being

While a variety of SWB indices were adopted by researchers in the past, three related yet distinct constructs—life satisfaction, happiness, and EWB—are among the most frequently posed criteria to reflect one’s overall assessment and experience of life [4,30,31,32,33]. Life satisfaction reflects more of a cognitive evaluation of one’s life; happiness draws more on emotions though still retaining cognitive, evaluative components; EWB captures the transient affective experience [34]. When studying the association between income and SWB, life satisfaction and happiness are most frequently adopted while most researchers have only studied one of the two, but not both [9,13]. In this study, we will test all three indices in most of our hypotheses. Studying all three indices vis-à-vis could not only enhance the external validity of our hypothesized relationships, but also allow for discovering differentiated effects of our focal constructs (financial satisfaction, a sense of control, IVR, and ASM) in influencing the cognitive versus emotional aspects of SWB.

### 2.2. The Mediation Mechanism of Financial Satisfaction

As described by the *Relativity Hypothesis*, people engage in a social comparative process when assessing their financial situations such that the impact of their relative income on SWB was found to matter more than the absolute amount [16,35,36,37,38]. Researchers have adopted financial satisfaction to reflect the subjective assessment of the discrepancy between their desirable financial resources and their actual financial resources [39]. People’s desires and expectations for financial resources are not only influenced by their actual needs but also by observing how other people financially meet their needs (i.e., standards of comparison). Abundant empirical evidence supports the hypothesis that one’s income and other financial resources work through subjective financial satisfaction to influence life satisfaction [9,12]. However, scarce research has tested the mediating roles of financial satisfaction in the link between income (and other financial resources) and two other indices of SWB—happiness and EWB. We posit that similar rationales for the mediating link (income→financial satisfaction→life satisfaction) could apply to happiness and EWB. Because happiness captures the hedonic aspect of SWB while EWB is more associated with a general sense of meaning [14], which is different from life satisfaction, we do not expect the same effect size. Nevertheless, we hypothesize the same direction (positive) between financial satisfaction and happiness as well as EWB. Thus,

**Hypothesis** **1.** *Financial satisfaction mediates the link between income and subjective well-being, including (a) life satisfaction, (b) happiness, and (c) EWB*.

### 2.3. The Mediation Mechanism of a Sense of Control

Another important mediator bridging the link between income and SWB is *a sense of control*. Lachman and Weaver [13] confirmed that income is positively related with both personal mastery and perceived constraints, the two dimensions of a sense of control. In addition, an extensive body of research links a sense of control with various positive life outcomes, including physical and psychological health [40,41,42]. The perceived sense of control in life has been frequently found to be positively associated with higher happiness [13,43], personal freedom [20,44], political freedoms [20,45,46], and tolerance [20]. Combining the two lines of research streams on the link between income and a sense of control and the link between a sense of control and positive life experiences, we hypothesize a positive, mediating role of a sense of control between income and SWB. Thus,

**Hypothesis** **2.** *A sense of control mediates the association between income and subjective well-being, including (a) life satisfaction, (b) happiness, and (c) EWB*.

### 2.4. Indulgence versus Restraint (IVR) Cultures

The direct, mediating, and moderating effects of culture on SWB promise great potential in the search for predictors of SWB beyond income [32,47,48]. However, little is known about how the newly added cultural dimension in Hostede’s framework [21,25], IVR, would directly influence SWB or moderate the mediating roles of financial satisfaction and a sense of control in the link between income and SWB.

As people in more indulgence cultures have already enjoyed much freedom to act as they please, to spend their disposable income, and to indulge in leisure [10], the additional impact of financial satisfaction on freeing up people’s desires should be weaker than those in more restraint cultures. In contrast, people in more restraint cultures would rely more on their satisfaction with financial resources before shopping for more non-necessity goods and services. Therefore, we posit weaker impacts of financial satisfaction on SWB for individuals in more indulgence cultures. In other words, people in more indulgence cultures do not need to rely as much on financial satisfaction to derive higher SWB as those in more restraint cultures.

Similarly, because people in indulgence cultures enjoy higher levels of personal control and lower levels of constraints than people in restraint cultures, the additional contributions of a sense of control to life satisfaction and happiness become less critical. Therefore, we also propose that positive impacts of a sense of control on SWB are weaker in more indulgence cultures than in more restraint cultures. Thus,

**Hypothesis** **3.** *Compared with people in more restraint cultures, people in more indulgence cultures have higher subjective well-being, including (a) life satisfaction and (b) happiness*.

**Hypothesis** **4a/4b.** *The impacts of financial satisfaction on subjective well-being, including (a) life satisfaction and (b) happiness, are weaker in more indulgence cultures than in more restraint cultures*.

**Hypothesis** **4c/4d.** *The impacts of a sense of control on subjective well-being, including (a) life satisfaction and (b) happiness, are weaker in more indulgence cultures than in more restraint cultures*.

### 2.5. Ability to Savor the Moment (ASM)

Bryant [26,49] proposes that people differ in their ability to savor along three temporal orientations: ability to derive pleasure by anticipating future positive events beforehand, ability to savor while a good event is happening, and ability to reminisce about past positive events afterwards. In our study, ASM is emphasized because the present temporal orientation more closely reflects people’s overall capacity to savor positive experiences than the other two temporal orientations, thus, it is more predictive for SWB in life. In addition, these three abilities are highly correlated with each other [26].

Bryant [27,49] has demonstrated that people’s self-evaluation of their ability to obtain and savor positive events (i.e., ASM) can promote well-being because people with higher ASM not only generate, intensify, and prolong more pleasure from positive outcomes in life, but also regulate, manipulate, and sustain positive feeling more effectively. The moderating roles of people’s ASM have not yet been discussed in the literature. We posit that individuals’ ASM would amplify the positive association between financial satisfaction and SWB for two reasons. First of all, because people with a higher ASM possess higher capacity to derive, regulate, and sustain pleasure from positive experiences in life, they would derive more positive emotions from the same level of satisfaction with financial situations than those with a lower ASM. Secondly, individuals with higher financial satisfaction tend to engage in more shopping and consumption activities and people with a higher ASM could further generate, intensify, and prolong more pleasure from the shopping and consumption activities [29,50]. Therefore, we posit the following,

**Hypothesis** **5.** *Individuals’ ASM is positively associated with their subjective well-being, including (a) life satisfaction, (b) happiness, and (c) EWB*.

**Hypothesis** **6.** *The impacts of financial satisfaction on subjective well-being, including (a) life satisfaction, (b) happiness, and (c) EWB, are stronger for individuals with a higher ASM than for those with a lower ASM*.

Note that we did not hypothesize the moderating effects of ASM on the mediating roles of a sense of control in the link between income and SWB because a sense of control generally is more related to cognitive senses of structure and order in life [11], which do not shift by individuals’ emotional regulation capabilities such as ASM.

**Figure 1:**
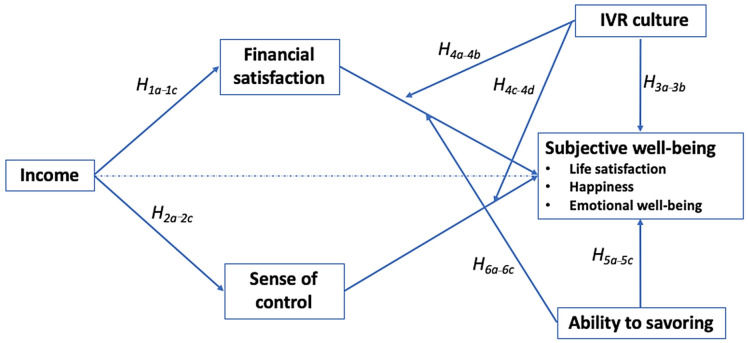
Conceptual model.

The full conceptual model is presented in Figure 1.

## 3. Materials and Methods

This paper used two studies to test the hypotheses. Study 1 tested cross-national level hypotheses with the World Values Survey (WVS) database (2017–2020). Study 2 tested individual level hypotheses with a survey in China.

### 3.1. Samples

In study 1, there were 49,097 participants from 38 countries and regions after eliminating invalid responses (some participants did not answer or were not asked) (see Appendix A). The mean age of the sample was 43.39 (SD = 16.19), and 47.6% of the sample was male. In study 2, 785 valid responses were used for final analyses after eliminating invalid responses such as who did not pass an attention check (i.e., an item that required them to select a certain response option). The mean age of the sample was 28.40 (SD = 6.54), and 47.8% of the sample was male.

### 3.2. Materials and Procedures

In study 1, the WVS was approved by the Institute for Comparative Survey Research Institutional Review Board. It has been used in many studies [51]. The survey includes various dimensions of attitudes toward democracy, covering countries with widely divergent cultures, from high indulgence (e.g., Australia) to high restraint cultures (e.g., China). We have matched the countries both in the list of World Values Survey (WVS) database (2017–2020) and Hofstede’s Cultural Dimensions website (https://geerthofstede.com). Thus, we had 38 countries and regions for the final statistical analysis. Please see Appendix A for more details.

In study 2, the data were collected on Wenjuanxing (www.wjx.cn), a survey platform that provides functions equivalent to Amazon’s Mechanical Turk in China [52], from January to February of 2019. This study purchased paid sample services from www.wjx.cn, and the sample services randomly distributed questionnaires to their qualified panels, who were employed by China’s firms. At the beginning of the survey, all participants were informed about the research project and provided written consent. After finishing the survey, all the participants received monetary credits from the sample service of Wenjuanxing. Eight hundred and eighty-six Chinese adults completed the study on Wenjuanxing (www.wjx.cn), one hundred and one participants did not pass an attention check (i.e., an item that required them to select a certain response option) and were removed. This study was approved by the Ethics Committee of Jinan University.

### 3.3. Measures

Income: In study 1, a single question was designed to capture respondents’ income levels based on their household income, including all wages, salaries, pensions, and other incomes. Participants provided their answers by using 10-point scales in the respective countries. In study 2, the respondent’s income level was measured based on their household income by using 8-point scales, from Level 1 for below 30,000 RMB to Level 8 for above 500,000 RMB.

Financial satisfaction (FS): In study 1, FS was measured by a single question described as: “How much are you satisfied with your family’s financial situation?” Participants provided their answers by using 10-point scales. In study 2, FS was measured by material affluence from Kasser and Sheldon’s [53] Material and Time Wealth Scale, which includes eight items such as “I have had enough money to buy what I need to buy”; participants provided their answers by using 5-point scales (from “1 = strongly disagree” to “5 = strongly agree”). Cronbach’s alpha of the scale was 0.76 in the present study.

A sense of control (SC): In study 1, SC was measured using a single question described as, “Some people feel they have completely free choice and control over their lives, while other people feel that what they do has no real effect on what happens to them.” Respondents were asked to rate their freedom in choosing and controlling their lifestyle by using 10-point scales. In study 2, we used the personal mastery subscale from Lanchman and Weaver’s [13] Sense of Control Scale to measure participants’ SC, which includes four questions, such as “What happens in my life is often beyond my control.” Responses were given on a 5-point scale from “1 = strongly disagree” to “5 = strongly agree”. Cronbach’s alpha of this scale was 0.66 in this study.

Life satisfaction (LS): In study 1, LS was measured by a single question described as, “All things considered, how satisfied are you with your life as a whole these days?” Participants provided their answers by using 10-point scales. In study 2, LS was measured by the Satisfaction with Life Scale [54], which includes five items rated on a 7-point scale from “1 = strongly disagree” to “7 = strongly agree”, such as “In most ways my life is close to my ideal.” In this study, the Cronbach’s alpha of the scale was 0.86.

Happiness: In study 1, it was measured by a single question described as, “Taking all things together, would you say you are…” Participants provided their answers by using 4-point scales. Reverse coding was performed in line with the concept. In study 2, happiness was measured by one question, “Do you generally feel happy?” Participants provided their answers by using a 5-point scale, from “1 = very unhappy” to “5 = very happy”.

Emotional well-being (EWB): EWB, which includes positive affect and negative affect, was measured by the Scale of Positive and Negative Experience (SPANE) [34] in study 2. This measure is a brief 12-item scale with 6 items (such as “Positive, Good, Pleasant, Happy, Joyful, Contented”) devoted to positive experience and 6 items (such as “Negative, Bad, Unpleasant, Sad, Afraid, Angry”) designed to assess negative experience. All of the items are answered on a 5-point scale ranging from “1 = very rarely or never” to “5 = very frequently or always”. Cronbach’s alpha was 0.77 for positive affect and 0.76 for negative affect in this study. The individual’s EWB was measured by subtracting the summed negative score from the summed positive score.

Indulgence versus Restraint (IVR): We tested the moderation effect of IVR in study 1; scores of Hofstede’s IVR dimension were used to represent cultural traits of different countries, with a higher indulgence index indicating that the country is more endowed with indulgence versus restraint orientations (see Appendix A). The data were obtained at Hofstede’s Cultural Dimensions website (https://geerthofstede.com).

Ability to savor the moment (ASM): We tested the moderation effect of ASM in study 2. Savoring the Moment scale was adopted from Bryant’s [26] Savoring Beliefs Inventory (SBI). The SBI measures the perception of one’s ability to feel pleasure through anticipating positive experiences, savoring positive moments as they occur, and reminiscing about past positive events [55]. We just used the Savoring the Moment Subscale to measure respondent’s ability to savor the moment, which includes eight items, such as “Find it easy to enjoy self when want to”; participants provided their answers by using the 5-point scale from “1 = strongly disagree” to “5 = strongly agree”. Cronbach’s alpha of the scale was 0.69 in the study.

Control variables: In study 1, gender, age, education, and immigration status were introduced as covariates to control for their effects on income, financial satisfaction, happiness, and IVR. In study 2, gender, age, and education were introduced to control for their effect on financial satisfaction, a sense of control, life satisfaction, happiness, EWB, and ASM.

Statistical analysis: Since core constructs in this study involve two levels—individuals and countries, a multilevel path analysis was used to analyze the moderated mediation model. We also used the Robust Maximum Likelihood Estimation for parameter estimation. Specifically, income, financial satisfaction, a sense of control, happiness, life satisfaction, gender, age, and education are at Level 1, while the indulgence–restraint indices are at Level 2. For testing the cross-level moderating effect, level 1 variables were group mean-centered, and level 2 variables were grand mean-centered [56]. The advantage of this two-level approach is that it does not only effectively avoid deviation caused by mixing within-group variance and between-group variance between variables, but also directly (rather than step by step) estimates the indirect effects of interactions between variables and coefficients that constitute the path to obtain more precise results. Mplus 7.0 was used for the main models. Meanwhile, because the indirect effects estimated by the above-mentioned two-level path analysis method often do not obey the normal distribution, we used Parametric Bootstrap to perform repeated sampling (both 20,000 repeated sampling) to generate confidence intervals of indirect effects, therefore more effectively testing the significance of indirect effects in the model and the significance of indirect effects under different moderation conditions. Thus, we also used R 4.0.3 (R Foundation for Statistical Computing, Vienna, Austria, 2020) for data analysis.

## 4. Results

### 4.1. Results of Study 1

#### 4.1.1. Descriptive Statistics

Descriptive statistics and correlation analysis are shown in Table 1. The results indicate that income was positively related to financial satisfaction, sense of control, life satisfaction, and happiness, and was negatively related to indulgence index. There were also positive relationships among financial satisfaction, sense of control, life satisfaction, and happiness.

#### 4.1.2. Confirmatory Factorial Analysis

Before testing the structural model, an assessment of the properties of the measurement model is required. We conducted a Confirmatory Factorial Analysis (CFA) [57], using Lisrel 8.80, to diagnose if common-method variance was a problem. Since indulgence index was an observable variable in the national level, it was not included in the CFA models. The results show that the hypothesized model including all five constructs (income, financial satisfaction, sense of control, life satisfaction, happiness) generated good fit indexes to data, i.e., CFI = 1.00, GFI = 1.00, CMIN/df = 4.71, RMSEA = 0.04, CMIN-*p* < 0.001. This was better than any other constructs models. According to these results, the common-method bias could be considered to have been overcome.

#### 4.1.3. Hypotheses Testing (DV = Life Satisfaction)

We first tested the mediation role of financial satisfaction and a sense of control between income and life satisfaction, as hypothesized in H1a. As shown in Table 2, both financial satisfaction and sense of control played a partial mediating role between income and life satisfaction. The indirect effect of financial satisfaction was significant (*B* = 0.17, 95% CI [0.30, 0.36]), and the indirect effect of sense of control was significant (*B* = 0.05, 95% CI [0.07, 0.09]). Thus, both H1a and H2a were supported.

In addition, the indulgence index was positively related to life satisfaction (*B* = 0.01, *p* < 0.05, see Model 3 in Table 2), meaning that compared with people in more restraint cultures, people in more indulgence cultures have higher life satisfaction, thus H3a was supported. H4a, proposing a moderating effect of IVR in the association between financial satisfaction and life satisfaction, was tested by assessing cross-level moderation effects of the IVR index on the random slope between financial satisfaction and life satisfaction. The IVR index had a negative impact on the random slope between financial satisfaction and life satisfaction (*B* = −0.002, *p* < 0.05, see Model 4 in Table 2).

**Figure 2:**
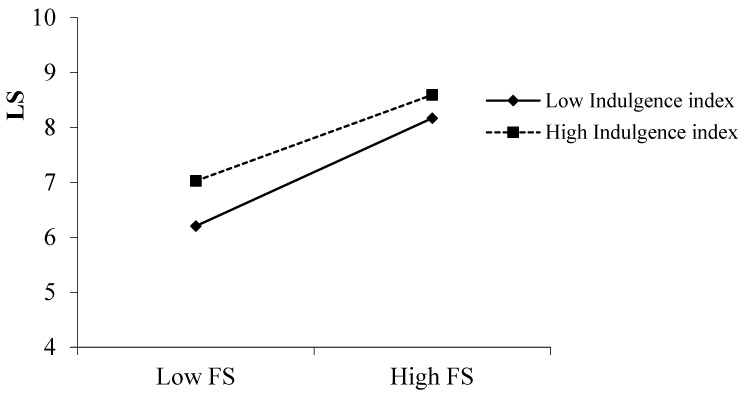
Interaction between Financial Satisfaction (FS) and Indulgence versus Restraint (IVR) in predicting Life Satisfaction (LS) in study 1.

Results of simple slope analyses (Cohen, 2003) are presented in Figure 2. When the IVR index was high (one standard deviation above the mean), financial satisfaction was positively related to life satisfaction (*B* = 0.37, *p* < 0.001); when the IVR index was low (one standard deviation below the mean), financial satisfaction was also positively related to life satisfaction (*B* = 0.48, *p* < 0.001), but the random slope between financial satisfaction and life satisfaction in low IVR was higher than high IVR (*B* = −0.11, *p* < 0.05, 95% CI [−0.19, −0.02]), indicating that the impacts of financial satisfaction on life satisfaction were weaker in more indulgence cultures than in more restraint cultures. Thus, H4a was supported. However, the moderated mediation effects of IVR were not significant for a sense of control (*B* = 0.001, *p* = 0.07), thus, H4c was not supported.

#### 4.1.4. Hypotheses Testing (DV = Happiness)

Similar procedures, as reported above, were used to test H1b, H2b, H4b, and H4d with happiness as dependent variable. As shown in Table 3, both financial satisfaction and sense of control were playing a partial mediating role between income and happiness. The indirect effect of financial satisfaction was significant (*B* = 0.03, 95% [0.05, 0.06]), and the indirect effect of sense of control was significant (*B* = 0.01, 95% CI [0.01, 0.02]). Hence, both H1b and H2b were supported.

In addition, the indulgence index was positively related to happiness (*B* = 0.003, *p* < 0.05, see Model 3 in Table 3), meaning that compared with people in more restraint cultures, people in more indulgence cultures have higher life satisfaction, thus H3b was supported. We also tested the moderation effect of IVR on the association between financial satisfaction and happiness (H4b), and the moderation effect of IVR on the association between sense of control and happiness (H4d); none of them was significant (*B*_1_ = 0.0003, *p* = 0.15; *B*_2_ = 0.0002, *p* = 0.83). Hence, both H4b and H4d were not supported.

### 4.2. Results of Study 2

#### 4.2.1. Descriptive Statistics

The means, standard deviation, and correlation of the variables in study 2 are presented in Table 4. The results indicate that income was positively related to financial satisfaction, sense of control, ability to savor, life satisfaction, positive affect, and emotional well-being, and was negatively related to negative affect. However, no significant relationship was found with happiness. There were also positive relationships among financial satisfaction, sense of control, ability to savor, life satisfaction, happiness, and emotional well-being.

#### 4.2.2. Confirmatory Factorial Analysis

Before testing the structural model, an assessment of the properties of the measurement model is required. We conducted a Confirmatory Factorial Analysis (CFA) by using the item parceling [58], using Lisrel 8.80, to diagnose if common-method variance was a problem. Since income was an observable variable, it was not included in the CFA models. The results show that the hypothesized model including all six constructs (financial satisfaction, sense of control, ability to savor the moment, positive affect, negative affect, life satisfaction) generated acceptable fit indexes to data, i.e., CFI = 0.91, GFI = 0.92, CMIN/df = 2.52, RMSEA = 0.04, CMIN-*p* < 0.001. This was better than any other constructs models. According to these results, the common-method bias could be considered to have been overcome.

#### 4.2.3. Testing Mediating Effects (DV = Life Satisfaction)

Firstly, after controlling for gender, age, and education, the results (see Table 5) supported the mediation roles of financial satisfaction between income and life satisfaction (*B* = 0.08, 95% CI [0.05, 0.12]). Similarly, a sense of control was also found to mediate the relationship between income and life satisfaction (*B* = 0.03, 95% CI [0.02, 0.05]). Therefore, both H1a and H2a were supported at the individual level. Furthermore, income was found to have direct, positive impact on life satisfaction (*B* = 0.10, *p* < 0.01) after introducing the two mediators. Therefore, financial satisfaction and sense of control were found to partially mediate the relationship between income and life satisfaction.

#### 4.2.4. Testing Mediating Effects (DV = Happiness)

We used the same approach to test the mediating effects of financial satisfaction and sense of control between income and happiness. The results (see Table 5) supported the hypothesis that the mediation role of financial satisfaction between income and happiness was significant (*B* = 0.02, 95% CI [0.004, 0.04]). The mediation role of sense of control between income and happiness was not significant (*B* = −0.001, 95% CI [−0.01, 0.01]). By controlling the mediators, the direct effect of income on happiness was not significant (*B* = 0.05, 95% CI [−0.02, 0.11]), indicating *full* mediation of financial satisfaction in the relationship between income and happiness. In sum, the results supported H1b but not H2b.

#### 4.2.5. Testing Mediating Effects (DV = EWB)

Similarly, the indirect effect of financial satisfaction between income and EWB was significant (*B* = 0.04, 95% CI [0.02, 0.07]). The indirect effect of sense of control in the positive link between income and EWB was also significant (*B* = 0.01, 95% CI [0.01, 0.03]). By controlling the mediators, the direct effect of income on EWB was not significant (*B* = 0.01, 95% CI [−0.05, 0.07]), indicating full mediation of financial satisfaction and a sense of control in the relationship between income and EWB; please see Table 5. Therefore, both H1c and H2c were supported.

#### 4.2.6. Ad hoc Analyses of Mediating Effects (DV = PA & NA)

As shown in Table 5, similar evidence was found for the full mediation roles of financial satisfaction and a sense of control in the link between income and positive affect (*p* < 0.01). However, no supportive evidence was found for the two mediation mechanisms for the link between income and negative affect. Therefore, it appears that the full mediation roles of both factors in the link between income and EWB (as in hypotheses H1c and H2c) were mainly attributable to their effects on positive affect, rather than negative affect.

#### 4.2.7. Examination of the Moderated Mediation Models (DV = Life Satisfaction)

We first tested H5a and H6a with life satisfaction as the dependent variable. In particular, H5a proposed a positive effect of ASM on life satisfaction, which was supported as shown in Table 6 (*B* = 0.23, *p* < 0.01). H6a proposed a moderation effect of ASM on the relationship between financial satisfaction and life satisfaction, and the result showed that an interaction between financial satisfaction and ASM on life satisfaction was significant (*B* = 0.24, *p* < 0.05). Thus, H6a was supported.

**Figure 3:**
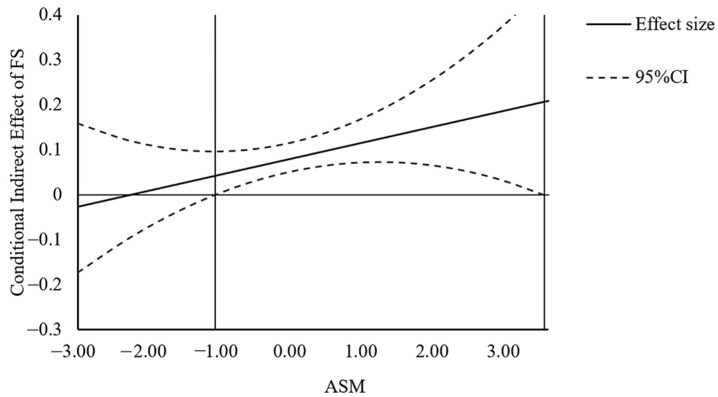
The indirect effect of Income on Life Satisfaction (LS) via Financial Satisfaction (FS) versus Ability to Savor the Moment (ASM), with confidence bands in study 2.

Furthermore, we used the Johnson–Neyman technique [59,60,61] to plot the conditional indirect effects. The conditional indirect effect of income on life satisfaction via financial satisfaction is shown in Figure 3. These results also supported H6a. 

#### 4.2.8. Examination of the Moderated Mediation Models (DV = Happiness)

As shown in Table 7, H5b proposed a positive direct impact of ASM on happiness, which was supported (*B* = 0.25, *p* < 0.01). However, our results showed that the interaction of ASM and financial satisfaction on happiness was not significant (*B* = 0.10, *p* = 0.27). Thus, H6b was not supported.

#### 4.2.9. Examination of the Moderated Mediation Models (DV = EWB)

As shown in Table 8, the results showed that ASM was not related to EWB (EWB) (*B* = 0.08, *p* = 0.28). Thus, H5c was not supported. However, our results showed that the interaction between financial satisfaction and ASM on EWB was significant (*B* = 0.25, *t* = 2.61, *p* < 0.01). Thus, H6c was supported. Meanwhile, we have also found that the interaction between sense of control and ASM on EWB was significant (*B* = 0.24, *t* = 2.89, *p* < 0.01), which indicates that ASM strengthened the effect of sense of control on EWB.

**Figure 4:**
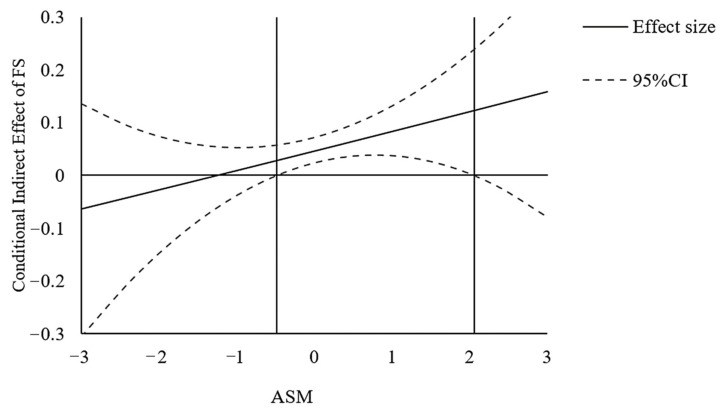
The indirect effect of Income on Emotional Well-being (EWB) via Financial Satisfaction (FS) versus Ability to Savor the Moment (ASM), with confidence bands in study 2.

**Figure 5:**
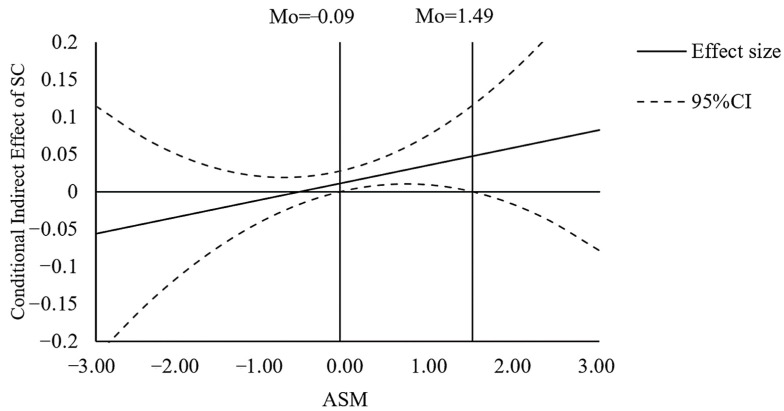
The indirect effect of Income on Happiness via Sense of Control (SC) versus Ability to Savor the Moment (ASM), with confidence bands in study 2.

Similarly, we used the Johnson–Neyman technique to plot the conditional indirect effects. The indirect effects of income on EWB via financial satisfaction (shown in Figure 4), and via sense of control (shown in Figure 5) are plotted with an accompanying 95% confidence band based on the second-order variance. These results also supported the H6c.

#### 4.2.10. Ad hoc Analyses of Moderated Mediation Models (DV = PA & NA)

As shown in Table 9, the results showed supportive evidence for direct and moderated roles of saving when examining positive affect (PA) separately. Specifically, ASM was found to be positively associated with individual PA (*B* = 0.15, *p* < 0.001). The significant positive interaction of financial satisfaction and ASM also indicated that ASM strengthened the effect of financial satisfaction on PA (*B* = 0.19, *t* = 3.49, *p* < 0.001). In addition, the interaction between a sense of control and ASM on PA was also significant (*B* = 0.12, *t* = 2.57, *p* < 0.05), which also indicated that ASM strengthened the effect of sense of control on PA.

The results of analyses with negative affect (NA) as the dependent variable are shown in Table 10. The results showed no support for the direct impact of ASM on NA (*B* = 0.08, *p* = 0.09). The interaction of financial satisfaction and ASM was not significantly related to NA (*B* = −0.06, *p* = 0.34). Thus, we find neither direct nor moderating effects of ASM when entering NA as an index of EWB. Even though the interaction between a sense of control and savoring was significant (*B* = −0.12, *p* < 0.05), the 95% bootstrap confidence interval, as presented in Table 10, indicated that the indirect effect of sense of control was not significant (*B* = −0.05, 95% CI [−0.03, 0.002]). 

### 4.3. General Discussion

#### Theoretical Contributions

With the two studies, we attempted to first examine the mediating roles of financial satisfaction and a sense of control in the link between income and SWB (life satisfaction, happiness, and EWB), and to further investigate the moderated mediation effects of national difference in IVR and individual difference in ASM. Our findings, as summarized in Appendix B, contribute to the SWB literature in at least three aspects.

First of all, we confirmed the existences of partial mediation mechanisms through which financial satisfaction and a sense of control bridge the link between income and SWB, indexed by life satisfaction and happiness at both cross-national and individual levels. While previous studies had only tested these two mediation mechanisms with individual samples from the United States [9,13], our study 1 contributes to the SWB literature by confirming both mediation mechanisms with a large scale of cross-national data, and expanding indices of SWB from a single index (life satisfaction) used in Johnson and Krueger [9] to both life satisfaction and happiness. Further, similar results were also found in study 2 using a national sample from China, which represents very different economic, social, and cultural contexts from the US, where the two previous studies were carried out. Our study 2 confirmed the partial mediation roles of financial satisfaction and a sense of control in the link between income and two SWB indices—life satisfaction and happiness. The consistent findings from our two studies seem to support mediation roles of both financial satisfaction and a sense of control in the link between income and life satisfaction (and happiness) besides direct impacts of income. However, our study 2 reported that financial satisfaction and a sense of control fully mediate the link between income and EWB, which is in contrast with the partial mediation relationships for the link between income and two other indices of SWB—life satisfaction and happiness. Our post hoc analyses further suggest such full mediation relationships hold true for both positive affect and negative affect.

The current SWB literature remains inconclusive regarding whether financial satisfaction and a sense of control fully or partially mediate the link between income and SWB. In previous studies, Lachman and Weaver [13] suggested a partial mediation of a sense of control, while Johnson and Krueger [9] asserted a full mediation of both financial satisfaction and a sense of control. Our cross-national study (i.e., study 1) and individual-level study (i.e., study 2) seemed to support Lachman and Weaver [13]’s argument in which SWB is measured with either cognitive or cognition-infused affective evaluations such as life satisfaction and happiness. However, people’s emotional well-being seems to fully rely on whether income can increase their financial satisfaction and their perceived sense of control. Otherwise, income increase may not lead to better emotional experiences in life.

The second theoretical contribution of our research is that we find direct, positive impacts of IVR cultural orientation on both life satisfaction and happiness, as well as its weakening effect on the mediation roles of financial satisfaction in the link between income and life satisfaction (but not happiness). As the newly developed dimension of cultural orientation in Hostede [25]’s framework, IVR has not yet received much attention in the SWB literature. Our findings obviously enrich the SWB literature by not only examining the direct impacts of IVR, but also investigating its moderating effects for the two mediation mechanisms. Interestingly, our study 1 reported that IVR seemed to weaken the positive impacts of financial satisfaction on life satisfaction, while such a weakening effect did not hold for happiness. In particular, we find that the mediation roles of financial satisfaction on happiness did not vary by IVR while the positive impacts of life satisfaction were weakened such that the positive association between financial satisfaction and life satisfaction in more indulgence cultures is not as strong as in more restraint cultures. Additionally, IVR was found to have no moderating effects on the mediating roles of a sense of control in the link between income and life satisfaction as well as the link between income and happiness. This finding seemed to suggest that a sense of control was a stable mediator to “transfer” income to SWB rather than financial satisfaction, at least at cross-national level.

Finally, we confirmed the direct, positive impact of ASM on both life satisfaction and happiness, but not on EWB. In particular, the positive impact of ASM on positive affect was significant, but was not significant on negative affect. Establishing the positive link between ASM on life satisfaction, happiness, and positive affect (but not negative affect) is of significant contribution to the SWB literature for two reasons. First of all, ASM, as a construct reflecting individuals’ ability to engage in self-indulgence, can be learned even in a more restraint culture. That provides an additional direction for a society to follow in order to improve overall life satisfaction and happiness. Secondly, we find that ASM is positively associated with positive affect. The positive association between ASM and positive affect seems to echo other researchers’ argument for the emotional values of self-indulgence. For example, self-indulgence was found to serve as a form of defense of self-integrity [62] and pursuit of self-worth [63] through the exploration of, and experimentation with, one’s own desire. However, our findings failed to support self-indulgence’s negative impacts. Instead, we find non-significance for the link between ASM and negative affect as previous studies proposed. The differential impacts of ASM on positive versus negative affects might explain why we failed to find an insignificant link between ASM and EWB; ASM only increases positive affect but does not decrease negative affect, thus it is not strong enough to significantly promote overall emotional well-being, similar to what Singh and Tripathi [50] found. Such intriguing findings appear to suggest that additional intervention mechanisms should be in place to decrease negative affect besides tapping into ASM to increase positive affect if a society strives for higher EWB for its members.

Individuals’ ASM does not only directly lead to a higher SWB, but also amplifies the mediation impacts of financial satisfaction in the link between income and life satisfaction and in the link between income and EWB. Compared with people with low ASM, individuals with high ASM seem to derive higher life satisfaction and EWB from financial satisfaction. However, we did not find such amplifier effects of ASM in the relationship between financial satisfaction and happiness. This finding further manifests how different aspects of SWB (cognitive versus emotional indices) might be impacted by income through varied mechanisms.

## 5. Limitations and Future Directions

The current research is not without limitations, so the findings and conclusions of our two studies should be considered in the light of these limitations. First of all, all SWB was measured in our study through self-reporting. Other measurement methods should be incorporated in future studies such as combining time use and activity-specific affect information adopted by other researchers to more accurately capture people’s affective experiences [22]. Further, the association between life circumstances such as income and SWB has been shown to be different by age and gender [22,23]. Whether our reported moderated mediation effects differ by age and gender warrants more research because our prescribed suggestions for increasing SWB through financial satisfaction and a sense of control, as well as the ASM and IVR orientations, would have to be adjusted if such gender and age differences exist.

## 6. Conclusions

The mediating effects of financial satisfaction and a sense of control in the link between income and SWB were supported at both national level and individual level. The mediating effects of financial satisfaction were found to be weaker in more indulgence cultures than in more restraint ones while the mediating effects of a sense of control remain the same. We also find that individuals’ ASM does not only directly lead to a higher SWB, but also amplifies the mediation impact of financial satisfaction in the link between income and life satisfaction and in the link between income and EWB.

## Figures and Tables

**Table 1:** Description and correlation analysis in study 1 (*N* = 49,097).

Variable	M ± SD	1	2	3	4	5	6	7	8
Level 2									
Indulgence index	46.27 ± 24.17								
Level 1									
1 Gender	-								
2 Immigrant or not	-	0.01							
3 Age	43.39 ± 16.19	−0.02 **	0.075 **						
4 Education level	-	0.01 **	0.04 ***	0.04 ***					
5 Income	4.65 ± 2.08	−0.03 ***	0.01 *	0.09 ***	0.02 **				
6 Financial satisfaction	6.17 ± 2.47	0.01	0.01	0.01	−0.05 ***	0.33 ***			
7 Sense of control	7.27 ± 2.28	−0.02 ***	−0.01 **	−0.03 **	−0.04 ***	0.13 ***	0.30 ***		
8 Life satisfaction	7.08 ± 2.29	0.02 ***	0.01 *	0.03 **	−0.04 ***	0.20 ***	0.58 ***	0.41 ***	
9 Happiness	3.14 ± 0.71	0.03 ***	−0.004	−0.05 **	−0.03 ***	0.14 ***	0.36 ***	0.25 ***	0.45 ***

Note. * *p* < 0.05, ** *p* < 0.01, *** *p* < 0.001. Gender: 1 = male (47.6%), 2 = female; Immigrant or not: 1 = not (94.5%), 2 = yes; Educational Level: 0 = early childhood education/no education (5.5%), 1 = primary education (13.2%), 2 = lower secondary education (17.3%), 3 = upper secondary education (25.0%), 4 = post-secondary non-tertiary education (8.8%), 5 = short-cycle tertiary education (7.5%), 6 = bachelor or equivalent (15.9%), 7 = master or equivalent (5.1%), 8 = doctoral or equivalent (1.7%), Income: ranges from 1 which indicates the lowest income group to 10 which indicates the highest income group in the country.

**Table 2:** Testing the moderated mediation effects for Life Satisfaction in study 1 (*N* = 49,097).

	The Dependent Variables	LS				FS	SC
The Independent Variables		Model 1	Model 2	Model 3	Model 4	Model 5	Model 6
Intercept _(_γ_00)_		7.02 ***	7.02 ***	7.02 ***	7.02 ***	6.09 ***	7.4 ***
Individual level (L1)							
Gender _(_γ_40)_		0.07 *	0.08 ***	0.04	0.09 ***	−0.01	−0.08 **
Age _(_γ_50)_		0.002	0.002	−0.001	0.08	0.002	−0.001
Education _(_γ_60)_		0.001	0.0001	0.01	0.001	0.001	0.08
Immigrant or not _(_γ_70)_		−0.09	−0.06	−0.13	0.002	−0.008	−0.004
Income _(_γ_10)_		0.23 ***	0.02 *		0.01	0.37 ***	0.14 ***
FS _(_γ_20)_			0.41 ***		0.42 ***		
SC _(_γ_30)_			0.26 ***		0.26 ***		
National level (L2)							
Indulgence index _(_γ_01)_				0.01 *	0.01 *		
Interaction							
FS× Indulgence index					−0.002 *		
SC × Indulgence index _(_γ_11)_					0.001		

Note. * *p* < 0.05, ** *p* < 0.01, *** *p* < 0.001. FS = Financial Satisfaction, SC = Sense of Control, LS = Life Satisfaction.

**Table 3:** Testing the moderated mediation effects for Happiness in study 1 (*N* = 49,097).

	The Dependent Variables	Happiness				FS	SC
The Independent Variables		Model 1	Model 2	Model 3	Model 4	Model 5	Model 6
Intercept _(_γ_00)_		3.13 ***	3.13 ***	3.13 ***	3.13 ***	6.09 ***	7.24 ***
Individual level (L1)							
Gender _(_γ_40)_		0.03 **	0.04 ***	0.03 **	0.04 ***	-0.01	−0.08 **
Age _(_γ_50)_		−0.002 *	−0.002 ***	−0.002 *	−0.002 **	0.002	−0.001
Education _(_γ_60)_		0.004	0.004 **	0.004	0.004 *	0.001	0.08
Immigrant or not _(_γ_70)_		−0.04	−0.02	−0.03	0.03	−0.01	−0.004
Income _(_γ_10)_		0.05 ***	0.01 ***		0.01 **	0.37 ***	0.14 ***
FS _(_γ_20)_			0.08 ***		0.08 ***		
SC _(_γ_30)_			0.04 ***		0.05 ***		
National level (L2)							
Indulgence index _(_γ_01)_				0.003 *	0.004 *		
Interaction							
FS × Indulgence index					0.0003		
SC × Indulgence index _(_γ_11)_					0.0002		

Note. * *p* < 0.05, ** *p* < 0.01, *** *p* < 0.001. FS = Financial Satisfaction, SC = A Sense of Control.

**Table 4:** Description and correlation analysis in study 2 (*N* = 785).

Variable	M	SD	1	2	3	4	5	6	7	8	9	10	11
1. Sex	-	-											
2. Age	28.40	6.54	−0.12 **										
3. Education level	-	-	0.06	−0.09 *									
4. Family annual income	2.70	1.32	−0.10 **	0.32 ***	0.27 ***								
5. Financial satisfaction	2.87	0.64	−0.00	0.07	0.15 ***	0.32 ***							
6. Sense of control	3.34	0.68	−0.05	−0.01	0.17 ***	0.20 ***	0.31 ***						
7. Ability to savor the moment	3.22	0.54	−0.01	−0.04	0.16 ***	0.13 ***	0.15 ***	0.43 ***					
8. Positive affect	3.31	0.58	−0.01	0.02	0.06	0.10 **	0.21 ***	0.18 ***	0.20 ***				
9. Negative affect	2.82	0.62	0.04	−0.12 **	−0.07	−0.10 **	−0.16 ***	−0.07	0.02	−0.39 ***			
10. Life satisfaction	4.17	1.14	0.03	0.08 *	0.17 ***	0.27 ***	0.41 ***	0.31 ***	0.24 ***	0.25 ***	−0.17 ***		
11. Happiness	3.18	0.97	−0.04	0.02	0.02	−0.07	0.05	0.08 *	0.03	0.65 ***	−0.25 ***	0.13 ***	
12. Emotional well-being	0.49	1.00	−0.03	0.09 *	0.07 *	0.12 ***	0.22 ***	0.15 ***	0.10 **	0.82 ***	−0.84 ***	0.25 **	0.54 ***

Note. * *p* < 0.05, ** *p* < 0.01, *** *p* < 0.001. Gender: 1 = male (47.8%), 2 = female; Educational Level: 1 = primary education (2.7%), 2 = lower secondary education (12.9%), 3 = upper secondary education (25.0%), 4 = bachelor or equivalent (50.2%), 5 = master or equivalent (4.8%), 6 = doctoral or equivalent (0.4%), Family annual income: 1 = ≤30,000 RMB (16.6%), 2 = 30,000~50,000 RMB (33.1%), 3 = 50,000~80,000 RMB (28.8%), 4 = 80,000~120,000 RMB (11.7%), 5 = 120,000~200,000 RMB (7.0%), 6 = 200,000~300,000 RMB (1.7%), 7 = 300,000~500,000 RMB (0.1%), 8 = >500,000 RMB (1.0%).

**Table 5:** Double mediation effect results in study 2.

	Effects		
	Estimate	CI_low_	CI_high_
Model 1: 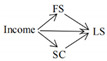	0.21	0.14	0.27
Direct effect1 (Income → LS)	0.10	0.03	0.16
Total indirect effect1	0.11	0.08	0.15
Income → FS → LS	0.08	0.05	0.12
Income → SC → LS	0.03	0.02	0.05
Model 2: 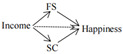	0.06	0.002	0.13
Direct effect2 (Income → Happiness)	0.05	−0.02	0.11
Total indirect effect2	0.02	0.002	0.04
Income → FS → Happiness	0.02	0.004	0.04
Income → SC → Happiness	−0.001	−0.01	0.01
Model 3: 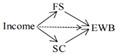	0.06	0.003	0.12
Direct effect3 (Income → EWB)	0.01	−0.05	0.07
Total indirect effect3	0.05	0.03	0.08
Income → FS → EWB	0.04	0.02	0.07
Income → SC → EWB	0.01	0.001	0.03
Model 4: 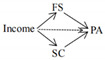	0.04	0.01	0.08
Direct effect4 (Income → PA)	0.01	−0.02	0.05
Total indirect effect4	0.03	0.02	0.05
Income → FS → PA	0.02	0.01	0.04
Income → SC → PA	0.01	0.003	0.02
Model 5: 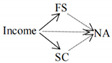	−0.02	−0.05	0.02
Direct effect5 (Income → NA)	0.004	−0.03	0.04
Total indirect effect5	−0.02	−0.04	−0.01
Income → FS → NA	−0.02	−0.04	−0.01
Income → SC → NA	−0.001	−0.01	0.01

Note. LS = Life Satisfaction, EWB = Emotional Well-being, PA = Positive Affect, NA = Negative Affect, FS = Financial Satisfaction, SC = Sense of Control; bootstrap resampling = 5000.

**Table 6:** Testing the moderated mediation effects for Life Satisfaction in study 2.

		Fit Indices		Bootstrap 95%			
Outcome		*R* ^2^	Lower	*β*	Lower	Upper	*t*
FS	Gender	0.11	23.21 ***	0.03	−0.06	0.12	0.70
	Age			−0.002	−0.01	0.01	−0.62
	Education			0.04	−0.01	0.09	1.61
	Income			0.15	0.12	0.19	8.26 ***
SC	Gender	0.06	11.83 ***	−0.06	−0.15	0.04	−1.22
	Age			−0.01	−0.02	0.001	−1.80
	Education			0.09	0.03	0.14	3.02 *
	Income			0.10	0.06	0.13	4.71 ***
LS	Gender	0.25	27.97 ***	0.12	−0.03	0.26	1.60
	Age			0.01	−0.01	0.02	1.12
	Education			0.07	−0.01	0.16	1.63
	Income			0.09	0.03	0.15	2.83 **
	FS			0.53	0.41	0.65	8.57 ***
	SC			0.26	0.14	0.38	4.23 ***
	ASM			0.23	0.09	0.37	3.13 **
	FS × ASM			0.24	0.04	0.43	2.41 *
	SC × ASM			−0.06	−0.23	0.11	−0.67

Note. * *p* < 0.05, ** *p* < 0.01, *** *p* < 0.001. FS = Financial Satisfaction, SC = Sense of Control, LS = Life Satisfaction, ASM = Ability to Savor the Moment.

**Table 7:** Testing the moderated mediation effects for Happiness in study 2.

		Fit Indices		Bootstrap 95%			
Outcome	Predictors	*R* ^2^	*F*	*β*	Lower	Upper	*t*
FS	Gender	0.12	21.47 ***	−0.04	0.09	0.16	−0.81
	Age			−0.002	−0.01	0.01	−0.67
	Education			0.02	−0.04	0.07	0.56
	Income1			0.06	0.03	0.09	3.61 ***
	Income			0.13	0.07	0.16	8.26 ***
SC	Gender	0.06	9.57 ***	0.06	−0.04	0.15	1.19
	Age			−0.01	−0.01	0.001	−1.80
	Education			0.08	0.02	0.14	2.70 *
	Income			0.10	0.06	0.13	4.71 ***
Happiness	Gender	0.05	3.85 ***	0.08	−0.06	0.21	1.10
	Age			0.002	−0.01	0.01	0.31
	Education			0.03	−0.05	0.11	0.72
	Income			0.04	−0.02	0.10	1.26
	FS			0.15	0.03	0.27	2.51 *
	SC			−0.07	−0.18	0.05	−1.12
	ASM			0.25	0.11	0.37	3.54 ***
	FS × ASM			0.10	−0.08	0.29	1.10
	SC × ASM			0.14	−0.02	0.30	1.67

Note. *p* * < 0.05, *p* *** < 0.001. FS = Financial satisfaction, SC = Sense of Control. Income = Household Income, ASM = Ability to Savor the Moment.

**Table 8:** Testing the moderated mediation effects for EWB in study 2.

		Fit Indices		Bootstrap 95%			
Outcome	Predictors	*R* ^2^	*F*	*β*	Lower	Upper	*t*
FS	Gender	0.11	23.21 ***	0.03	−0.06	0.12	0.70
	Age			−0.002	−0.01	0.01	−0.62
	Education			0.04	−0.01	0.09	1.61
	Income			0.15	0.12	0.19	8.26 ***
SC	Gender	0.06	11.83 ***	−0.06	−0.15	0.04	−1.22
	Age			−0.01	−0.02	0.001	−1.80
	Education			0.09	0.03	0.14	3.02 *
	Income			0.10	0.06	0.13	4.71 ***
EWB	Gender	0.10	9.31 ***	−0.04	−0.18	0.10	−0.59
	Age			0.01	−0.001	0.02	1.74
	Education			0.03	−0.05	0.11	0.68
	Income			0.002	−0.06	0.06	0.07
	FS			0.30	0.18	0.42	5.03 ***
	SC			0.14	0.02	0.26	2.36 *
	ASM			0.08	−0.06	0.22	1.09
	FS × ASM			0.25	0.06	0.43	2.61 **
	SC × ASM			0.24	0.08	0.41	2.89 **

Note. * *p* < 0.05, ** *p* < 0.01, *** *p* < 0.001. FS = Financial satisfaction, SC = Sense of Control, EWB = Emotional Well-being, ASM = Ability to Savor the Moment.

**Table 9:** Testing the moderated mediation effects for Positive Affect in study 2.

		Fit Indices		Bootstrap 95%			
Outcome	Predictors	*R* ^2^	*F*	*β*	Lower	Upper	*t*
FS	*Gender*	0.11	23.21 ***	0.03	−0.06	0.12	0.70
	*Age*			−0.002	−0.01	0.01	−0.62
	*Education*			0.04	−0.01	0.09	1.61
	*Income*			0.15	0.12	0.19	8.26 ***
SC	*Gender*	0.06	11.83 ***	−0.06	−0.15	0.04	−1.22
	*Age*			−0.01	−0.02	0.001	−1.80
	*Education*			0.09	0.03	0.14	3.02 *
	*Income*			0.10	0.06	0.13	4.71 ***
PA	*Gender*	0.12	11.36 ***	−0.004	−0.08	0.08	−0.10
	*Age*			−0.001	−0.01	0.01	−0.04
	*Education*			−0.01	−0.06	0.04	−0.49
	*Income*			0.01	−0.03	0.04	0.30
	*FS*			0.16	0.09	0.23	4.57 ***
	*SC*			0.09	0.02	0.15	2.51 ***
	*ASM*			0.15	0.07	0.23	3.75 ***
	*FS × ASM*			0.19	0.08	0.30	3.49 ***
	*SC × ASM*			0.12	0.03	0.22	2.57 *

Note. * *p* < 0.05, *** *p* < 0.001. FS = Financial satisfaction, SC = Sense of Control, PA = Positive Affect, ASM = Ability to Savor the Moment.

**Table 10:** Testing the moderated mediation effects for Negative Affect in study 2.

		Fit Indices		Bootstrap 95%			
Outcome	Predictors	*R* ^2^	*F*	*β*	Lower	Upper	*t*
FS	Gender	0.11	23.21 ***	0.03	−0.06	0.12	0.70
	Age			−0.002	−0.01	0.01	−0.62
	Education			0.04	−0.01	0.09	1.61
	Income			0.15	0.12	0.19	8.26 ***
SC	Gender	0.06	11.83 ***	−0.06	−0.15	0.04	−1.22
	Age			−0.01	−0.02	0.001	−1.80
	Education			0.09	0.03	0.14	3.02 *
	Income			0.10	0.06	0.13	4.71 ***
NA	Gender	0.06	5.11 ***	0.04	−0.05	0.12	0.84
	Age			−0.01	−0.02	−0.003	−2.78
	Education			−0.04	−0.09	0.01	−1.52
	Income			0.003	−0.04	0.04	0.168
	FS			−0.14	−0.22	−0.07	−3.80 **
	SC			−0.05	−0.13	0.02	−1.45
	ASM			0.08	−0.01	0.16	1.69
	FS × ASM			−0.06	−0.17	0.06	−0.95
	SC × ASM			−0.12	−0.22	−0.01	−2.23 *

Note. * *p* < 0.05, ** *p* < 0.01, *** *p* < 0.001. FS = Financial satisfaction, SC = Sense of Control, NA = Negative Affect, ASM = Ability to Savor the Moment.

## Data Availability

The raw data supporting the conclusions of this article will be made available by the authors, without undue reservation.

## Appendix A. Participants from Countries and Regions, and Scores of Hofstede’s IVR Dimension Used for Study 1

**Code of Countries and Regions in WAS**	**Countries and Regions (Participants)**	**Indulgence-Restraint**
20	Andorra (836)	65
32	Argentina (844)	62
36	Australia (1564)	71
50	Bangladesh (1131)	20
76	Brazil (1354)	59
152	Chile (827)	68
156	China’s mainland (2864)	24
158	Taiwan of China (1160)	49
170	Colombia (1514)	83
196	Cyprus (743)	70
231	Ethiopia (1147)	46
276	Germany (1334)	40
300	Greece (1016)	50
344	Hong Kong of China (2023)	17
360	Indonesia (3080)	38
364	Iran (1429)	40
368	Iraq (1087)	17
392	Japan (914)	42
400	Jordan (1103)	43
410	Korea South (1245)	29
417	Kyrgyz Rep (1056)	39
458	Malaysia (1308)	57
484	Mexico (1675)	97
554	New Zealand (624)	75
566	Nigeria (1082)	84
586	Pakistan (1521)	0
604	Peru (1262)	4
608	Philippines (1196)	42
630	Puerto Rico (1051)	90
642	Romania (886)	20
643	Russia (1353)	20
688	Serbia (870)	28
704	Vietnam (1151)	35
716	Zimbabwe (1120)	28
764	Thailand (1378)	45
804	Ukraine (885)	14
818	Egypt (1065)	4
840	U.S.A. (2399)	68
Note: 156 China’s mainland, 158 Taiwan of China, 344 Hong Kong of China, all of they are parts of the People’s Republic of China, here just used for data analyzed.		

## Appendix B. Summary of Hypotheses Testing Results

		**Cross-National Level**		**Individual Level**				
		Life satisfaction	Happiness	Life satisfaction	Happiness	EWB	PA	NA
H1a	income → financial satisfaction → life satisfaction	YES		YES				
H1b	income → financial satisfaction → happiness		YES		YES			
H1c	income → financial satisfaction → EWB					YES	YES	YES
H2a	income → a sense of control → life satisfaction	YES		YES				
H2b	income → a sense of control → happiness		YES		YES			
H2c	income → a sense of control → EWB					YES	YES	YES
H3a	IVR → life satisfaction	YES						
H3b	IVR → happiness		YES					
H4a	IVR → weaker H1a	YES						
H4b	IVR → weaker H1b		NO					
H4c	IVR → weaker H2a	No						
H4d	IVR → weaker H2b		NO					
H5a	ASM → life satisfaction			YES				
H5b	ASM → happiness				YES			
H5c	ASM → EWB					NO	YES	NO
H6a	ASM → stronger H1a			YES				
H6b	ASM → stronger H1b				NO			
H6c	ASM → stronger H1c					YES	YES	NO
Note: YES means “supported” while NO means “not supported”.								

## Appendix C. The Scales Adopted

**Scale 1:** Sense of Control (SC) [13]

1. I can do just about anything I really set my mind to.
2. When I really want to do something‚ I usually find a way to succeed at it.
3. Whether or not I am able to get what I want is in my own hands.
4. What happens in my life is often beyond my control.

**Scale 2**: Financial Satisfaction (FS) [53]

1. I have had enough money to buy the things that are important to me.
2. There has not been enough money to go around.
3. I have been able to buy what I want.
4. I have felt like I’m pretty poor.
5. My bank account has been too low.
6. I have had enough money to buy what I need to buy.
7. I have been broke.
8. I have had plenty of spare money.

**Scale 3:** Satisfaction with Life (SL) [54]

1. In most ways my life is close to my ideal.
2. The conditions of my life are excellent.
3. I am Satisfied with my life.
4. So far I have gotten the important things I want in life.
5. If I could live my life over, I would change almost nothing.

**Scale 4:** Emotional Well-being (EWB) [34]:

1. Positive
2. Negative
3. Good
4. Bad
5. Pleasant
6. Unpleasant
7. Happy
8. Sad
9. Afraid
10. Joyful
11. Angry
12. Contented

**Scale 5:** Ability to Savor Moment (ASM) [26]

1. Know how to make the most of good time.
2. Find it hard to hang onto a good feeling.
3. Can prolong enjoyment by own effort.
4. Am own ‘worst enemy’ in enjoying.
5. Feel fully able to appreciate good things.
6. Can’t seem to capture joy of happy moments.
7. Find it easy to enjoy self when want to.
8. Don’t enjoy things as much as should.
